# Controlling Nutritional Status Score as a Predictive Marker of In-hospital Mortality in Older Adult Patients

**DOI:** 10.3389/fnut.2021.738045

**Published:** 2021-09-20

**Authors:** Chengyu Liu, Mingwei Zhu, Xin Yang, Hongyuan Cui, Zijian Li, Junmin Wei

**Affiliations:** Department of General Surgery, Beijing Hospital, National Center of Gerontology, Institute of Geriatric Medicine, Chinese Academy of Medical Sciences, Beijing, China

**Keywords:** nutrition, controlling nutritional status, in-hospital mortality, elderly, malnutrition

## Abstract

The controlling nutritional status (CONUT) score assesses nutritional status and is associated with short- and long-term prognoses in some diseases, but the significance of the CONUT score for the prediction of in-hospital mortality in older adults is unknown. The purpose was to determine the importance of the CONUT score for the prediction of in-hospital mortality, short-term complications, length of hospital stay, and hospital costs in older adults. Our retrospective cohort study analyzed data from 11,795 older adult patients from two multicenter cohort studies. We performed receiver operating characteristic curve analysis using in-hospital mortality as the endpoint and determined the appropriate CONUT score cut-off by the Youden index. The patients were divided into two high and low groups according to the CONUT cut-off value, and the differences in clinical characteristics and in-hospital clinical outcomes between the two groups were compared. We compared the accuracy of the CONUT score and other nutrition-related tools in predicting in-hospital mortality by calculating the area under the receiver operating characteristic curve and performed univariate and multivariate analyses of predictors of in-hospital mortality. Among all the patients, 178 (1.5%) patients experienced in-hospital death. The optimal cut-off values was 5.5 for the CONUT score. The high CONUT group had a higher incidence of short-term complications and prolonged hospital stay than the low CONUT group (CONUT score <6), but hospital costs were not significantly higher. The CONUT score had the highest predictive ability for in-hospital mortality among the five nutrition-related parameters compared. Multivariate analysis showed that a high CONUT score (CONUT score ≥ 6) was an independent predictor of in-hospital mortality. In conclusion, the present study demonstrated that the CONUT score could be used to predict in-hospital mortality in older adults.

## Introduction

Older adult inpatients have a high incidence of nutritional risk and malnutrition (1). Malnutrition is one of several negative predictors that affect the risk of some diseases in hospitalized patients and can predict adverse clinical outcomes (2, 3). Current guidelines recommend that routine nutritional screening of all older adult inpatients should include screening for malnutrition with a validated tool to identify malnourished patients (4, 5). There is currently no gold standard method for diagnosing malnutrition, and many tools need to be applied to assess nutritional status (6).

The Controlling Nutritional Status (CONUT) score is a nutritional scoring tool that is calculated using serum albumin, total cholesterol level, and total lymphocyte count (7). Current studies suggest that CONUT is associated with short- and long-term prognoses in some diseases, particularly cancers (8–14). However, the predictive role of the CONUT score for in-hospital mortality in older adult inpatients has not been clearly established.

The purpose of this study was to investigate the prognostic role of the CONUT score on in-hospital mortality, in-hospital complications, length of hospital stay (LOS), and hospital costs in older adult inpatients, and to compare the predictive ability of CONUT scores with the Nutritional Risk Screening-2002 (NRS-2002), the Onodera Prognostic Nutritional Index (OPNI), the Instant Nutritional Assessment (INA), and the Geriatric Nutritional Risk Index (GNRI) for in-hospital mortality.

## Methods

### Participants

Data from 11,795 older adult patients in this retrospective cohort analysis were obtained from two multicenter cohort studies. The first study was a multicenter nutritional survey of consecutively admitted older adult inpatients at 14 hospitals in China from March to May 2012. The study protocol was approved by the Ethics Committee of Beijing Hospital (registration number: LLKYPJ2012002A). The other study was a multicenter study conducted in 34 hospitals in China from June to September 2014. The protocol was approved by the Ethics Committee of Beijing Hospital (registration number: 2014BJYYEC-022-02) and registered in the China Clinical Trial Registry (Registered No. ChiCTR-EPC-14005253). The inclusion criteria included: (1) age ≥ 65 years old, (2) conscious, and (3) no emergency surgery. Exclusion criteria included: (1) emergency patients, (2) refusal to participate and sign informed consent, and (3) missing information on in-hospital deaths.

### Data Collection

The following admission parameters were collected: age, gender, height, weight, body mass index (BMI), calf circumference (CC), upper arm circumference, handgrip strength, and laboratory data. Laboratory variables included leukocyte count, total lymphocyte count (TLC), hemoglobin, albumin, total protein, total bilirubin (TBIL), alanine aminotransferase (ALT), triglycerides (TG), total cholesterol (TC), blood urea nitrogen (BUN), and serum creatinine (Cr).

BMI <18.5 (if <70 years) or <20 (if >70 years) was considered low BMI (6). Low CC was defined as CC <34 cm in men and CC <33 cm in women (15). Low handgrip strength referred to handgrip strength <28.0 kg in men and <18.0 kg in women (15, 16).

### Nutrition-Related Tools

The NRS-2002 scores include three components: impaired nutritional status, disease severity, and age ≥70 years, with ≥ 3 indicating nutritional risk (17).

CONUT score = serum albumin score + TC score + TLC score (7). Serum albumin score (0, ≥ 3.5 g/dL; 2, 3.0–3.49 g/dL; 4, 2.50–2.99 g/dL; 6, <2.50 g/dL), TC score (0, ≥180 mg/dL; 1, 140–179 mg/dL; 2, 100–139 mg/dL; 3, <100 mg/dL), and TLC score (0, ≥1.6 109 [G]/L; 1, 1.20–1.59 g/L; 2, 0.80–1.19 g/L; 3, <0.8 g/L) (7).

INA grades were classified into four grades: 1, albumin ≥ 3.5 g/dl and TLC ≥ 1.5 109/L; 2, albumin ≥ 3.5 g/dl and TLC <1.5 109/L; 3, albumin <3.5 g/dl and TLC ≥ 1.5 109/L; 4, albumin <3.5 g/dl and TLC <1.5 109/L (18, 19).

OPNI = 10 × serum albumin (g/L) + 5 × TLC (109/L) (20, 21).

GNRI = 1.489 × serum albumin (g/L) + 41.7 × actual weight (kg)/ideal weight (kg) (22). The ideal weight was calculated with the Lorentzian formula (22, 23).

### Clinical Outcomes

Clinical outcome data were derived from a medical record system that included in-hospital mortality, short-term complications, LOS, and hospital costs. The short-term complications were defined as morbidity that occurred during hospitalization, and the occurrence of complications was judged according to the Clavien-Dindo classification system (24).

### Statistical Analysis

**Figure 1:**
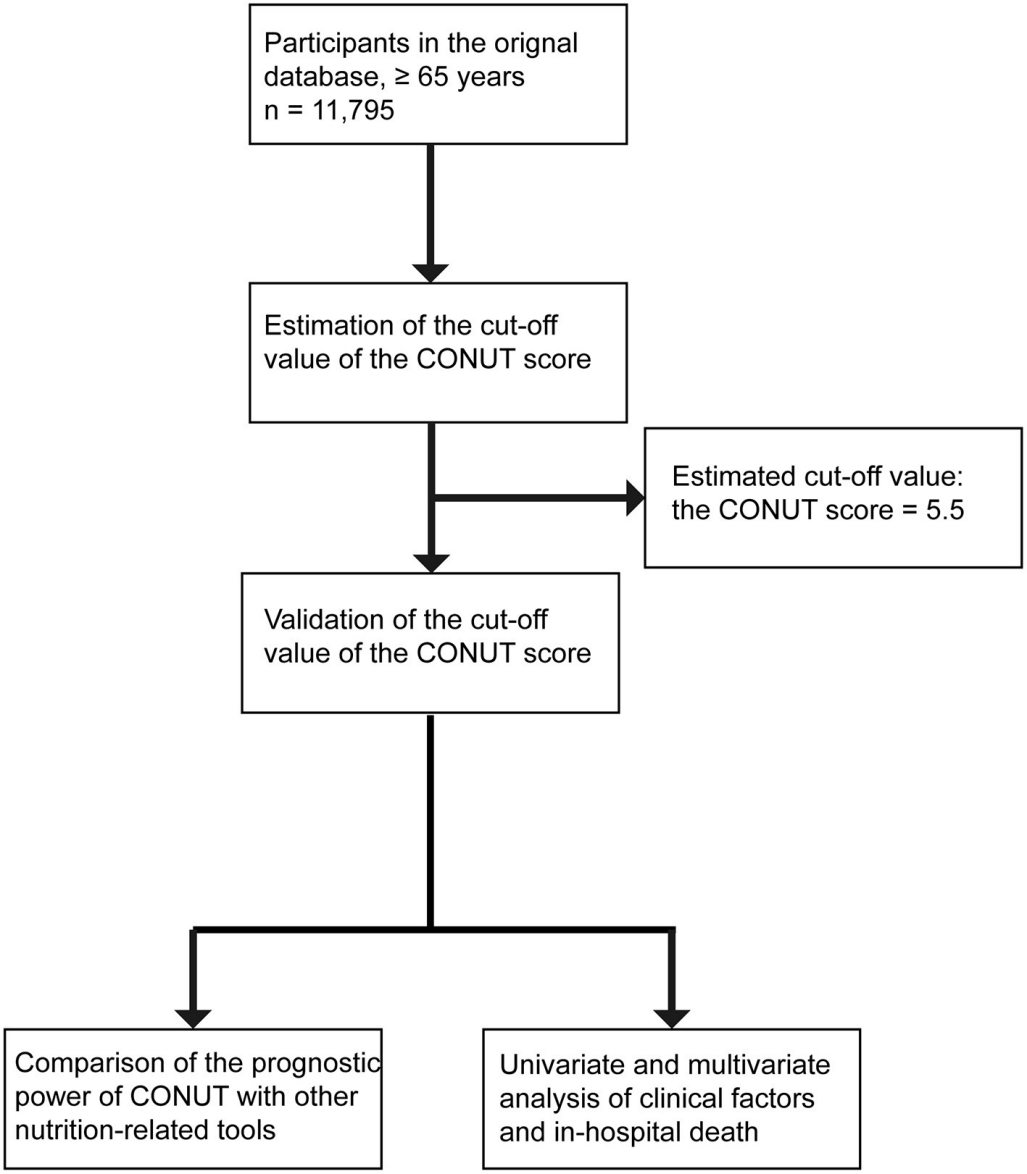
Flow chart of the study.

We performed receiver operating characteristic (ROC) curve analysis using in-hospital mortality as the endpoint and calculated the Youden index to determine the appropriate cut-off value for the CONUT score (Figure 1). Patients were then divided into two groups according to the cut-off value, and the differences in clinical characteristics and in-hospital clinical outcomes between the two groups were compared. The Shapiro-Wilk test was used to determine the type of distribution of the quantitative variables and the homogeneity of variance was assessed by F test or Levene test. Continuous variables were expressed as means ± standards deviation and were analyzed by t-text or Mann-Whitney U test. Categorical variables were expressed as numbers (percentages) and were analyzed by the chi-squared test (χ2). The accuracy of NRS-2002, CONUT, INA, OPNI, and GNRI values in predicting in-hospital mortality was compared by calculating the area under the curve (AUC). Univariate logistic regression (enter) was used to calculate odds ratios (ORs) and 95% confidence intervals (CI) between clinical factors and in-hospital mortality, and multivariate logistic regression (forward: likelihood ratio) was performed for statistically significant categorical variables in univariate logistic regression analysis. Hypothesis testing of the model was performed using the log-likelihood ratio test, and the Hosmer-Lemeshow test was used to test the goodness of fit of the statistical model. All statistical analyses were conducted using SPSS version 25, and a two-sided *P* value < 0.05 was considered significant.

## Results

### Patient Characteristics

The mean age of the enrolled inpatients was 74.8 ± 7.0 years, and 7,092 (60.1%) were male. Baseline characteristics of the patients included in the study shown in Table 1. Of all patients, there were 178 (1.5%) in-hospital deaths and 5,564 (47.2%) patients were at nutritional risk.

**Table 1:** Demographic and clinical characteristics of older adult patients.

**Characteristic**	**Total**	**CONUT score**		
	**(*n* = 11,795)**	** <6 (*n* = 5,930)**	**≥6 (*n* = 2,349)**	***P* value**
Age, year	74.8 ± 7.0	76.5 ± 7.5	74.8 ± 7.0	<0.001
Gender male	7,092 (60.1%)	1,558 (66.3%)	3,421 (57.7%)	<0.001
Height	164.0 ± 8.0	164.5 ± 8.1	163.6 ± 8.1	<0.001
Weight	62.5 ± 11.7	60.3 ± 12.1	63.8 ± 11.4	<0.001
BMI, kg/m^2^	23.2 ± 3.8	22.2 ± 3.8	23.8 ± 3.6	<0.001
CC, cm	32.1 ± 3.9	30.8 ± 4.1	32.8 ± 3.8	<0.001
Upper arm circumference, cm	25.7 ± 3.7	24.4 ± 3.8	26.5 ± 3.5	<0.001
Handgrip strength, kg	17.8 ± 16.5	13.6 ± 14.0	18.9 ± 16.7	<0.001
Total leukocyte count, 10^9^/ L	8.98 ± 123.88	9.76 ± 124.18	9.58 ± 152.11	0.959
TLC, 10^9^/ L	2.09 ± 4.22	1.04 ± 2.86	2.65 ± 5.00	<0.001
Hemoglobin, g/L	123.35 ± 33.44	112.94 ± 34.69	128.20 ± 27.70	<0.001
Albumin, g/dL	3.74 ± 0.55	3.30 ± 0.53	3.93 ± 0.41	<0.001
Total protein, g/mL	6.51 ± 1.58	6.64 ± 1.08	6.09 ± 1.41	<0.001
ALT, U/L	25.33 ± 50.16	27.85 ± 48.35	22.75 ± 39.40	<0.001
TBIL, umol/L	15.62 ± 28.20	16.60 ± 34.01	13.74 ± 18.99	<0.001
TG, mmol/L	1.54 ± 2.84	1.30 ± 1.85	1.64 ± 2.83	<0.001
TC, mg/dL	78.84 ± 31.22	69.92 ± 24.02	82.18 ± 32.53	<0.001
BUN, mmol/L	7.38 ± 19.00	8.88 ± 28.65	7.16 ± 18.18	0.007
Cr, umol/L	86.48 ± 140.80	95.89 ± 100.53	83.43 ± 148.43	<0.001

*BMI, Body mass index; CC, Calf circumference; TLC, Total lymphocyte count; ALT, Alanine aminotransferase; TBIL, Total bilirubin; TG, Triglycerides; TC, Total cholesterol; BUN, Blood urea nitrogen; Cr, Creatinine*.

### Estimation and Validation of the CONUT Score Cut-Off Value

**Figure 2:**
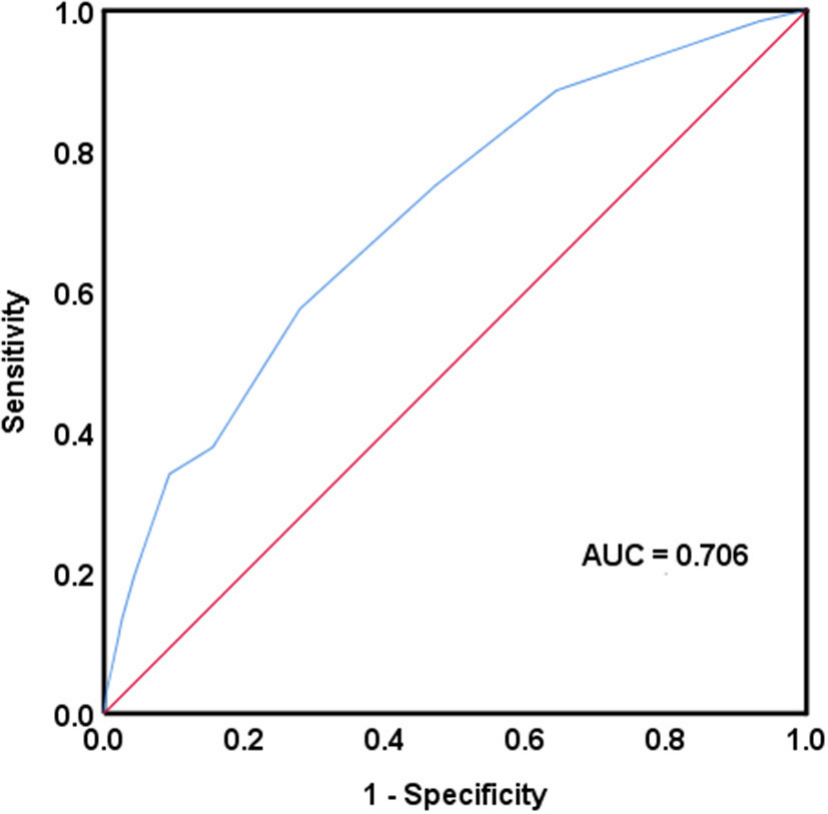
Receiver operating characteristic curve analysis was used to assess the accuracy of the controlling nutritional status score in predicting in-hospital mortality. AUC, area under the curve.

The CONUT score was calculated in 8,279 (70.2%) cases and it was missing in 3,516 (29.8%) cases. The median CONUT score was 4 (3, 6). By ROC curve analysis, the cutoff value at the maximum Youden index was 5.5, with a sensitivity of 57.6% and a specificity of 72.1% (Figure 2). The enrolled patients were divided into two groups, a low CONUT group (CONUT score <6; 5,930 patients, 50.3%) and a high CONUT group (CONUT score ≥6; 2,349 patients, 19.9%). Of 132 in-hospital deaths with complete CONUT scores, 56 (42.4%) were in the low CONUT group and 76 (57.6%) were in the high CONUT group.

There were significant differences in the following indicators between the two groups: age, gender, BMI, CC, upper arm circumference, handgrip strength, TLC, hemoglobin, albumin, total protein, TBIL, ALT, TG, TC, BUN, and Cr (*P* < 0.05).

The high CONUT group had a significantly higher incidence of short-term complications than the low CONUT group (11.0 vs. 6.4%, *P* < 0.001), and the LOS was prolonged (14.88 ± 9.96 vs. 13.84 ± 9.45 days, *P* < 0.001), but hospital costs were not significantly higher (4,415.84 ± 37,877.41 vs. 3,592.66 ± 5,350.06 EUR, *P* = 0.105).

### Comparison of the Predictive Ability of the CONUT Score With Other Nutrition-Related Tools

The CONUT score (AUC = 0.706) had the highest predictive ability for in-hospital mortality among the five nutrition-related tools compared, followed by the OPNI (AUC = 0.694). The NRS-2002 had the last predictive ability (AUC = 0.649) (Table 2).

**Table 2:** Comparison of AUC of different nutrition-related tools in predicting in-hospital mortality.

	**AUC**	***P* value**	**95%CI**
CONUT	0.706	<0.001	0.661–0.750
OPNI	0.694	<0.001	0.654–0.735
INA	0.653	<0.001	0.612–0.694
GNRI	0.653	<0.001	0.592–0.714
NRS-2002	0.649	<0.001	0.603–0.694

*AUC, Area under the curve; CI, Confidence Interval; CONUT, Controlling Nutritional Status; OPNI, Onodera Prognostic Nutritional Index; INA, Instant Nutritional Assessment; GNRI, Geriatric Nutritional Risk Index; NRS-2002, Nutritional Risk Screening-2002*.

### Univariate and Multivariate Analyses of Clinical Factors and In-hospital Death

Univariate logistic regression analysis indicated that in-hospital mortality was associated with gender, age, low BMI, low CC, low handgrip strength, low hemoglobin, low serum albumin, low total protein, low TC, nutritional risk, high INA, low GNRI, and high CONUT score (≥6) (Table 3). Multivariate analysis further showed that high CONUT score (≥ 6) (OR 3.242, 95% CI 2.148–4.892, *P* < 0.001), nutritional risk (OR 1.580, 95% CI 1.038–2.403, *P* = 0.33), and low handgrip strength (OR 2.116, 95% CI 1.168–3.834, *P* = 0.13) were independent risk factors for in-hospital mortality, −2 log-likelihood value was the smallest, and the goodness of fit test *P* was 0.251.

**Table 3:** Univariate logistic regression analysis of risk factors associated with in-hospital death in older adult patients (*n* = 11795).

**Characteristics**	**OR**	**95%CI**	***P* value**
Gender male	0.601	0.434–0.834	0.002
Age	1.088	1.067–1.109	<0.001
Total leukocyte count	1.000	0.998–1.002	0.928
TLC	0.891	0.784–1.103	0.078
Hemoglobin	0.988	0.983–0.994	<0.001
ALT	1.000	0.997–1.003	0.951
TBIL	1.003	1.000–1.007	0.079
TG	0.830	0.686–1.003	0.053
TC	0.991	0.984–0.999	0.022
BUN	1.002	0.997–1.007	0.419
Cr	1.000	1.000–1.001	0.180
CONUT	1.400	1.305–1.502	<0.001
OPNI	0.970	0.940–1.001	0.061
INA	1.605	1.404–1.836	<0.001
GNRI	0.937	0.920–0.955	<0.001
NRS-2002	1.442	1.319–1.576	<0.001
Low BMI	2.421	1.587–3.694	<0.001
Low CC	2.304	1.596–3.326	<0.001
Low handgrip strength	2.433	1.527–3.876	<0.001
Nutritional risk	2.403	1.750–3.299	<0.001
CONUT≥6	3.507	2.475–4.970	<0.001

*OR, Odds Ratio; TLC, Total lymphocyte count; ALT, Alanine aminotransferase; TBIL, Total bilirubin; TG, Triglycerides; TC, Total cholesterol; BUN, Blood urea nitrogen; Cr, Creatinine; CONUT, Controlling Nutritional Status; OPNI, Onodera Prognostic Nutritional Index; INA, Instant Nutritional Assessment; GNRI, Geriatric Nutritional Risk Index; NRS-2002, Nutritional Risk Screening-2002; BMI, Body mass index; CC, Calf circumference*.

## Discussion

Our study indicated that CONUT score can be used as a predictive tool for in-hospital mortality in older adult patients, and older adult patients with high CONUT score have a higher risk of in-hospital death and complications as well as hospital length of stay.

The CONUT score is simple and easy to obtain and is calculated using objective values. The CONUT score is a nutrition-related tool that includes serum albumin, TC, and TLC (7). Studies suggest that all three components of the CONUT score are associated with short-term prognosis that encompasses in-hospital mortality in older adult patients. Low serum albumin levels are associated with increased short-term and long-term mortality in hospitalized patients (25, 26), and serum albumin levels are an important predictor of in-hospital mortality or in-hospital complications in older adult patients (27, 28). The increased mortality predicted by albumin may be explained by two main mechanisms. First, albumin has specific antioxidant functions because of its structure, and hypoalbuminemia may result in cellular oxidative damage and apoptosis (29). Second, serum albumin levels report on the state of systemic protein metabolism and inflammation (27). Although it is controversial whether serum albumin levels directly indicate malnutrition, studies show that declining serum albumin levels serve as a marker of inflammation associated with nutritional risk and the risk of developing adverse clinical outcomes (30, 31).

Most studies suggest that low TC is associated with a higher risk of death in older adult patients (32–35). Low TC levels represent deterioration of nutritional status and exacerbated inflammation in older adult patients (32, 36, 37). A TLC less than 0.8 G/L was associated with the risk of in-hospital death, readmission, and LOS in hospitalized patients (38). A low TLC was also associated with higher mortality in older adult patients and was a predictor of prognosis (39, 40). Reduced TLC is also associated with decreased immune status and inflammatory status (41–43).

We found that the CONUT cut-off value was effective in distinguishing older adult inpatients with and without adverse outcomes. Older adult inpatients with CONUT scores higher than 6 had a higher risk of in-hospital death, a higher risk of short-term complications, and a longer LOS. Multivariate logistics regression analysis indicated that a CONUT score greater than 6 could be used as an independent risk factor for in-hospital mortality in older adult patients. Although current studies support that the CONUT score has a predictive effect on the prognosis of patients with a variety of diseases, the current findings have different cut-off values (10, 12, 14). Therefore, the predictive role of different CONUT score cut-off values need to be validated in populations with different diseases, and the best cut-off value should be confirmed in multicenter, large-sample, prospective clinical studies in the future.

According to the results of our study, in comparison with NRS-2002, INA, OPNI and GRNI in predicting in-hospital mortality, the CONUT score had the highest AUC value in ROC curve analysis, indicating a better predictive value of the CONUT score. Sze et al. showed that the CONUT score was slightly more discriminating for short-term prognosis than the OPNI and GNRI in patients with chronic heart failure (44). Consistent with our findings, the CONUT score has good prognostic value compared with other simple nutrition-related tools.

This study has several strengths. Our study is the first to the best of our knowledge to investigate the short-term prognostic value of the CONUT score in older adult inpatients. The predictive value of higher CONUT score on admission is an important finding, which enables clinicians to identify older adult patients at risk of death who may benefit from interventions such as early nutritional supplementation more quickly.

This study has several limitations. First, the results of our study cannot explain causality because this was a retrospective study combining two multicenter studies, and the two studies have some heterogeneity. Second, potential factors affecting immune nutritional status, such as acute stress status, cancer-related inflammation, chronic renal failure, and cirrhosis, were not obtained. Additionally, serum albumin levels as an indicator of nutritional status are unreliable for patients with recent acute stress; excluding older adult patients with acute stress will allow for a more accurate predictive effect of the CONUT score. Third, no information on whether surgery or chemotherapy was performed was available. Finally, we only assessed in-hospital clinical outcomes, so it is difficult to draw any conclusions about long-term prognosis.

In summary, the CONUT score has high prognostic accuracy and has advantages in predicting in-hospital mortality in older adult patients. The findings of this study indicate that high CONUT scores were associated with an increased risk of in-hospital mortality, short-term complications, and longer hospital stays in older adult patients. The CONUT score could be used as a simple nutrition-related tool for predicting in-hospital mortality in older adult patients.

## Data Availability Statement

The raw data supporting the conclusions of this article will be made available by the authors, without undue reservation.

## Ethics Statement

The studies involving human participants were reviewed and approved by the Ethics Committee of Beijing Hospital. The patients/participants provided their written informed consent to participate in this study.

## Author Contributions

CL and MZ conceptualized, analyzed, and interpreted data, and wrote the manuscript. XY, HC, and ZL collected, and interpreted the clinical data, and revised the manuscript critically for important content. JW interpreted the clinical data, and revised the manuscript critically for important content. All authors read and approved the final manuscript criteria.

## Conflict of Interest

The authors declare that the research was conducted in the absence of any commercial or financial relationships that could be construed as a potential conflict of interest.

## Publisher's Note

All claims expressed in this article are solely those of the authors and do not necessarily represent those of their affiliated organizations, or those of the publisher, the editors and the reviewers. Any product that may be evaluated in this article, or claim that may be made by its manufacturer, is not guaranteed or endorsed by the publisher.
